# Secretory Microneme Proteins Induce T-Cell Recall Responses in Mice Chronically Infected with Toxoplasma gondii

**DOI:** 10.1128/mSphere.00711-18

**Published:** 2019-02-27

**Authors:** Iti Saraav, Qiuling Wang, Kevin M. Brown, L. David Sibley

**Affiliations:** a Department of Molecular Microbiology, Washington University School of Medicine, St. Louis, Missouri, USA; Stanford University

**Keywords:** antigen-specific T cells, gamma interferon detection assays, memory T cells, microneme antigens, recall response, toxoplasmosis

## Abstract

Microneme (MIC) proteins play important roles in the recognition, adhesion, and invasion of host cells by Toxoplasma gondii. Previous studies have shown that MIC proteins are highly immunogenic in the mouse and recognized by human serum antibodies. Here we report that T. gondii antigens MIC1, MIC3, MIC4, and MIC6 were capable of inducing memory responses leading to production of gamma interferon (IFN-γ) by T cells from T. gondii-infected mice. Production of IFN-γ was demonstrated using enzyme-linked immunosorbent spot (ELISPOT) assay and also intracellular cytokine staining. All four MIC antigens displayed very high sensitivity (100%) and specificity (86 to 100%) for detecting chronic infection. Interestingly, IFN-γ was produced by both CD4^+^ and CD8^+^ T cells in BALB/c mice but primarily by CD4^+^ T cells in C57BL/6 mice. Phenotypic characterization of IFN-γ-producing CD4^+^ and CD8^+^ T cells in BALB/c mice and CD4^+^ T cells in C57BL/6 mice revealed effector memory T cells (CD44^hi^ CD62L^lo^) as the predominant cells that contributed to IFN-γ production in response to MIC antigens. Effector memory responses were seen in mice of different major histocompatibility complex class II (MHC-II) haplotypes, suggesting that MIC antigens contain epitopes that are broadly recognized.

**IMPORTANCE** Current diagnosis of toxoplasmosis relies almost exclusively on antibody detection, and while detection of IgG provides a useful estimate of prior infection, it does not alone indicate immune status. In contrast, detection of IFN-γ responses to T. gondii antigens has been used to monitor immune responsiveness in HIV-infected patients, thus providing valuable predictions about the potential for disease reactivation. However, specific T. gondii antigens that can be used in assays to detect cellular immunity remain largely undefined. In this study, we examined the diagnostic potential of microneme antigens of T. gondii using IFN-γ detection assays. Our findings demonstrate that MIC antigens (MIC1, MIC3, MIC4, and MIC6) elicit IFN-γ responses from memory T cells in chronically infected mice. Monitoring IFN-γ production by T cells stimulated with MIC antigens provided high sensitivity and specificity for detection of T. gondii infection in mice. Taken together, these studies suggest that microneme antigens might be useful as an adjunct to serological testing to monitor immune status during infection.

## Abstract (precis)

Current diagnosis of toxoplasmosis relies almost exclusively on antibody detection, and while detection of IgG provides a useful estimate of prior infection, it does not alone indicate immune status. In contrast, detection of IFN-γ responses to T. gondii antigens has been used to monitor immune responsiveness in HIV-infected patients, thus providing valuable predictions about the potential for disease reactivation. However, specific T. gondii antigens that can be used in assays to detect cellular immunity remain largely undefined. In this study, we examined the diagnostic potential of microneme antigens of T. gondii using IFN-γ detection assays. Our findings demonstrate that MIC antigens (MIC1, MIC3, MIC4, and MIC6) elicit IFN-γ responses from memory T cells in chronically infected mice. Monitoring IFN-γ production by T cells stimulated with MIC antigens provided high sensitivity and specificity for detection of T. gondii infection in mice. Taken together, these studies suggest that microneme antigens might be useful as an adjunct to serological testing to monitor immune status during infection.

## INTRODUCTION

Toxoplasma gondii is an obligate intracellular protozoan parasite that infects a wide range of warm-blooded hosts and causes toxoplasmosis. The infection is typically acquired through exposure to soil, food, or water that is contaminated with oocysts (containing sporozoites) or ingestion of undercooked meat containing viable tissue cysts (containing bradyzoites) (1, 2). Infection is characterized by an acute phase, in which parasites (i.e., sporozoites or bradyzoites) cross the intestinal epithelium, differentiate to tachyzoites that migrate to draining lymph nodes, and widely disseminate throughout the body. The acute infection is resolved by the development of protective immune responses. The acute phase is followed by chronic infection, characterized by cysts containing bradyzoites in the skeletal muscle and central nervous system of the infected host (3). Usually, T. gondii infection in healthy individuals is clinically asymptomatic. However, the infection can be serious in several circumstances, including for immunocompromised patients, who risk reactivation of chronic infection, and for naive women during pregnancy, in whom infection can lead to congenital infection (4, 5).

Cell-mediated immunity plays a crucial role in host resistance to T. gondii infection (6). In response to T. gondii infection, interleukin 12 (IL-12) signaling by macrophages and dendritic cells stimulates T cells and natural killer (NK) cells to produce gamma interferon (IFN-γ) (7, 8). IFN-γ is a major regulator of cell-mediated immunity which activates hematopoietic and nonhematopoietic effector cells to control parasite replication (9–12). During *Toxoplasma* infection in the mouse, CD8^+^ T cells are thought to be the major effector cells, while CD4^+^ T cells play a supportive role (13, 14). CD8^+^ T cells can both produce IFN-γ and kill infected cells, while CD4^+^ T cells contribute to control by IFN-γ secretion (15). It is primarily the production of IFN-γ and not perforin-mediated cytolytic activity by CD8^+^ T cells that is required for protection against T. gondii infection (16). Memory T cells are critical for long-term protection against T. gondii. CD4^+^ and CD8^+^ memory T cells are essential for the control of T. gondii proliferation and prevent reactivation of disease (17–20). There are two primary subsets of these long-lived T cells, known as central memory (Tcm) and effector memory (Tem) T cells. Tcm cells mainly reside in secondary lymphoid organs, express high levels of lymphoid homing molecules such as CCR7 and CD62L, and readily differentiate into effector cells in response to antigen. Tem cells are primarily present in nonlymphoid organs, do not express CCR7 and CD62L, and display immediate effector function (21, 22). One of the hallmarks of memory T cells is the capacity to mount an enhanced and potent recall response through T-cell receptor recognition of cognate antigen loaded on major histocompatibility complex (MHC) molecules of antigen-presenting cells. This response is critical for long-term immunity but can also be exploited for diagnostic detection of pathogens using purified microbial antigens.

In T. gondii, there are three main secretory compartments, called dense granules (GRA proteins), rhoptries (ROP), and micronemes (MIC proteins), which release proteins during or after host cell invasion (23, 24). Microneme secretion occurs constitutively at low levels but is upregulated in response to environmental factors such as contact with host cells or elevated intracellular calcium (23, 25, 26). The host cell invasion process of T. gondii is initiated by the interaction of the proteins released from micronemes with host cell receptors, primarily based on binding to carbohydrates (23, 24). For example, MIC1, MIC4, and MIC6 are known to form a complex that exerts an important role in host cell invasion (27, 28). We have previously shown that bovine serum albumin (BSA) combined with the phosphodiesterase inhibitor zaprinast induced microneme secretion in a protein kinase G-dependent manner and that this pathway was further augmented by elevation of intracellular Ca^2+^ (29). Excretory secretory antigens (ESA) of T. gondii are known for their high immunogenicity in different experimental models, and these antigens can induce protective immunity mediated by both antibody- and cell-dependent mechanisms (30–32). Several microneme proteins, such as MIC1, MIC3, MIC4, and MIC6, have been shown to be potential vaccine candidates based on studies in the murine model of toxoplasmosis (33–35). Although it has been shown that immunization with MIC1 and MIC4 confers protection against oral infection with ME49 in mice (33, 35), the mechanism of this protective effect is not known.

In this study, we examined memory T-cell responses to MIC antigens (MIC1, M2AP, MIC3, MIC4, MIC6, and MIC10) and examined the phenotypes of these cells during chronic infection. We report that MIC antigens (MIC1, MIC3, MIC4, and MIC6) induce memory T-cell recall responses leading to production of IFN-γ during chronic infection with T. gondii. Phenotypic analysis revealed that primarily CD4^+^ but also CD8^+^ effector memory T cells that recognized MIC antigens were maintained in mice chronically infected with T. gondii. These findings suggest that MIC antigens (MIC1, MIC3, MIC4, and MIC6) have potential as diagnostic markers for cell-mediated responses that could be used to augment existing serological approaches.

## RESULTS

### Expression and purification of endotoxin-free recombinant microneme proteins.

To study immunological responses to *Toxoplasma*, we cloned and purified several of the major protein components present in ESA. MIC proteins were expressed in Escherichia coli as soluble His-tagged proteins fused to Sumo, a bacterial ubiquitin-like modifying protein that often confers solubility on partner proteins. MIC6, M2AP, and MIC10 were expressed as full-length proteins, while MIC3, MIC4, and MIC1 were expressed as truncated proteins to enhance their expression and solubility (Table 1). The soluble recombinant proteins were purified by Ni affinity chromatography. SDS-PAGE analysis of the purified recombinant proteins showed that they migrated with the predicted molecular mass of each protein construct together with the Sumo tag (Table 1). The lower band of 12.5 kDa (migrates to 23 kDa) observed in some of these protein preparations likely represents a breakdown product of Sumo (Table 1). Endotoxin-contaminated proteins used in immunological assays can initiate strong responses due to engagement of Toll-like receptor 4 (TLR4) (36, 37). Therefore, the proteins were treated with endotoxin removal resin containing modified polylysine affinity ligand. After treatment, endotoxin levels of purified MIC proteins were <0.1 endotoxin unit (EU)/mg, as determined by a *Limulus* assay (Table 1). An endotoxin level below 1 EU/mg (<0.1 ng/mg) is considered a safe limit, as it typically does not interfere with assays (38, 39).

**TABLE 1:** Endotoxin levels of the MIC proteins after polymyxin B treatment

Protein	Molecular wt, full length (kDa)	No. of amino acids in construct	Mol wt (kDa) of:		Endotoxin level (EU/ml)	
			Tested fragment	Tested fragment with Sumo	Before treatment	After treatment
MIC1	48.6	20–340	35.2	58.2	2.878	0.051
M2AP	34.6	22–330	34.6	57.6	3.114	0.066
MIC3	40.5	134–383	27.4	50.2	3.209	0.048
MIC4	63	58–231	19.0	42	3.212	0.068
MIC6	36.7	23–349	36.7	59.7	3.003	0.053
MIC10	23.1	1–198	23.1	46.2	2.602	0.043
Sumo	12.5	1–100	12.5		3.124	0.071

### Detection of MIC antigens using IFN-γ ELISPOT assay.

**FIG 1:**
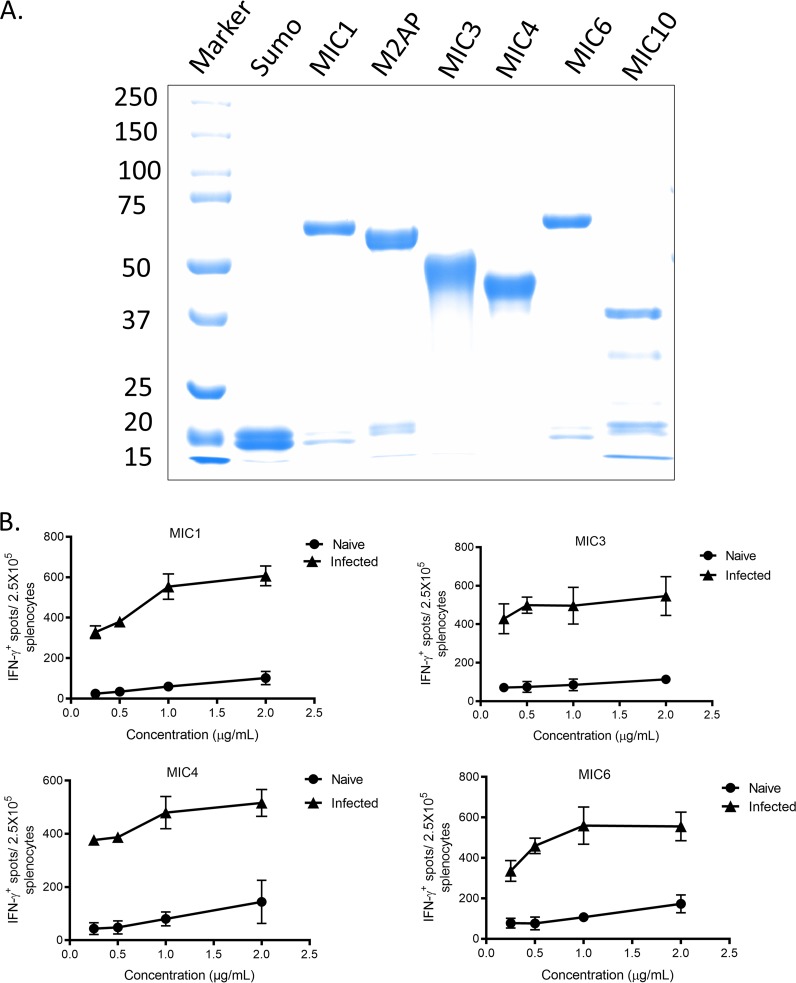
Separation of purified MIC fusion proteins by SDS-PAGE. (A) Coomassie blue-stained gel of recombinant proteins. Sumo was used as a control for the fusion proteins. The smaller bands in the recombinant protein lanes likely represent Sumo (12.5 kDa) as a breakdown product. Values on the left are molecular masses, in kilodaltons. (B) Dose-response curves to identify optimal concentration of MIC proteins for ELISPOT assay. Splenocytes from C57BL/6 mice that were naive (*n* = 3) or chronically infected with T. gondii (*n* = 3) were stimulated with different concentrations of MIC proteins for 24 h. For each protein, concentrations that resulted in the maximum positive response in infected mice with minimal responses in naive mice were selected. Data are presented as mean of IFN-γ^+^ spots/2.5 × 10^6^ cells ± SD from three mice in one experiment.

**FIG 2:**
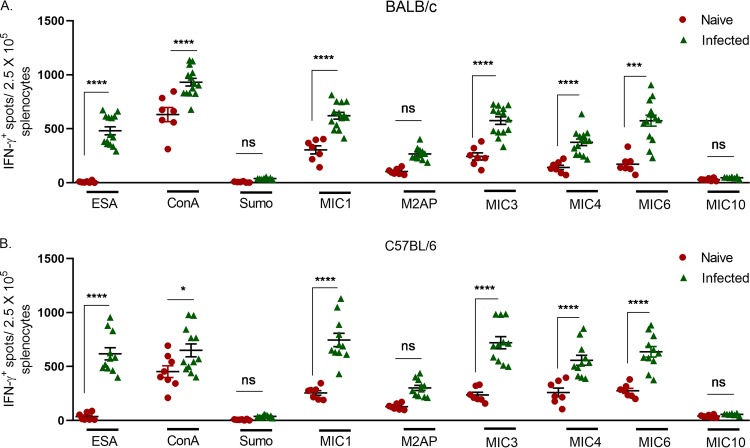
ELISPOT assay of IFN-γ production by splenocytes from mice that were naive or chronically infected with T. gondii. Mouse splenocytes were collected 30 days after T. gondii infection and stimulated with ESA as an antigen-specific positive control (1 µg/ml), ConA as a nonspecific positive control (1 µg/ml), Sumo as a negative control (1 µg/ml), or MIC1, M2AP, MIC4, MIC6, or MIC10 (1 µg/ml), or MIC3 (0.5 µg/ml). Cells were stimulated for 24 h from either BALB/c (A) or C57BL/6 (B) mice. ELISPOT analysis was performed on 6 naive and 12 chronically infected BALB/c mice and 7 naive and 11 chronically infected C57BL/6 mice. Ordinary one-way ANOVA with Sidak’s multiple-comparison test was used to compare ELISPOT results. *, *P ≤ *0.05; ***, *P ≤ *0.001; ****, *P ≤ *0.0001. ns, nonsignificant.

To identify whether T cells from T. gondii-infected mice can respond to MIC antigens *ex vivo*, splenocytes obtained from naive mice and mice chronically infected with T. gondi were stimulated with purified MIC antigens. Optimal concentrations of MIC protein (MIC1, MIC3, MIC4, and MIC6) were determined using different doses to perform an IFN-γ enzyme-linked immunosorbent spot (ELISPOT) assay (Fig. 1B). Concentrations of 1 µg/ml for MIC1, MIC4, and MIC6 and 0.5 µg/ml for MIC3 were selected, as they resulted in positive responses in infected mice and lower responses in naive mice (Fig. 1B). To evaluate two different MHC haplotypes, experiments were conducted with both BALB/c mice (H-2^d^ haplotype) and C57BL/6 mice (H-2^b^ haplotype). We observed that both BALB/c and C57BL/6 chronically infected mice produced statistically significantly higher levels of IFN-γ than naive mice when stimulated with MIC antigens (MIC1, MIC3, MIC4, and MIC6) (Fig. 2). In contrast, no significant response was seen in IFN-γ levels of BALB/c and C57BL/6 chronically infected mice when stimulated with M2AP and MIC10 (Fig. 2). ESA was used as a positive control, and stimulation resulted in significantly higher production of IFN-γ in CD4^+^ T cells from T. gondii-infected mice than from naive mice (Fig. 2). As expected, the mitogen concanavalin A (ConA) stimulated responses in both groups of animals (Fig. 2). We did not observe any response to Sumo, confirming that positive response observed in the ELISPOT assay was specific to MIC antigens and not due to underlying endotoxin contamination. Receiver operating characteristic (ROC) curve analyses are typically used to determine whether an assay fulfills the criterion for a reliable diagnostic test based on relative sensitivity and specificity (40, 41). We performed ROC analyses to evaluate the specificity and sensitivity of MIC antigens in the ELISPOT assay. MIC antigens that gave positive responses in the ELISPOT assay were used for ROC analyses. In the IFN-γ ELISPOT assay, MIC1, MIC3, MIC4, and MIC6 had a specificity of 100%, and sensitivity ranged from 86 to 100% in both strains of mice (Table 2). These findings indicate that MIC antigens (MIC1, MIC3, MIC4, and MIC6) may provide a useful diagnostic tool for detection of IFN-γ secretion during chronic infection.

**TABLE 2:** Specificity and sensitivity values for the MIC proteins based on ELISPOT assays using ROC curves

Protein	C57BL/6^*a*^			BALB/c^*b*^		
	Cutoff	Specificity (%)	Sensitivity (%)	Cutoff	Specificity (%)	Sensitivity (%)
MIC1	>359	100	100	> 408	100	100
MIC3	>416	100	100	>398	100	93
MIC4	>398	100	88	>234	100	93
MIC6	>402	100	88	>312	100	86

a C57BL/6 mice included 7 naive and 11 infected mice.

b BALB/c mice included 6 naive and 12 infected mice.

### Detection of IFN-γ production by intracellular cytokine staining.

**FIG 3:**
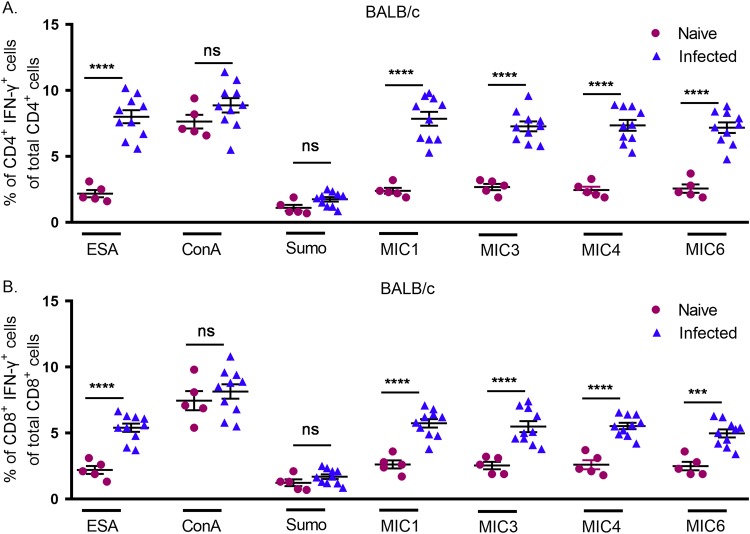
Intracellular cytokine staining of IFN-γ production by CD4^+^ and CD8^+^ T cells from BALB/c mice. Splenocytes were obtained from mice that were naive or chronically infected with T. gondii and stimulated with ESA (1 µg/ml), ConA (1 µg/ml), SUMO (1 µg/ml), MIC1, MIC4, or MIC6 (1 µg/ml), or MIC3 (0.5 µg/ml) for 24 h. The experiment was performed on 5 naive and 10 chronically infected mice. Ordinary one-way ANOVA with Sidak’s multiple-comparison test was used to compare results between naive and infected cells.

To characterize T. gondii-specific CD4^+^ and CD8^+^ T-cell responses, splenocytes from naive and chronically infected mice were examined by intracellular staining for IFN-γ. Figure S1A in the supplemental material shows the gating strategy used for the intracellular staining for IFN-γ. In splenocytes from infected mice BALB/c mice stimulated with ESA, ∼14% of CD3^+^ IFN-γ^+^ cells were CD4^+^ T cells, while ∼4% were CD8^+^ T cells (Fig. S1B). Stimulation of splenocytes with MIC antigens resulted in significant increases in IFN-γ production by both CD4^+^ and CD8^+^ T cells from infected mice in comparison to that in naive mice, but CD4^+^ T cells produced greater amounts of IFN-γ than CD8^+^ T cells (Fig. 3). As expected, stimulation with ConA triggered IFN-γ production in both naive and infected mice (Fig. 3).

10.1128/mSphere.00711-18.1 FIG S1 IFN-γ intracellular staining by splenocytes following *in vitro* stimulation. (A) Gating strategy for evaluation of CD4^+^ and CD8^+^ T cells producing IFN-γ. Figure represents IFN-γ intracellular staining from splenocytes obtained from BALB/c mice chronically infected with T. gondii when stimulated with 1 µg/ml of MIC1 protein for 24 h. (B) Percentages of CD4^+^ and CD8^+^ T cells among CD3^+^ IFN-γ^+^ splenocytes isolated from C57BL/6 and BALB/c mice stimulated with ESA or ConA. Download FIG S1, TIF file, 0.6 MB. Copyright © 2019 Saraav et al. 2019 Saraav et al. This content is distributed under the terms of the Creative Commons Attribution 4.0 International license.

**FIG 4:**
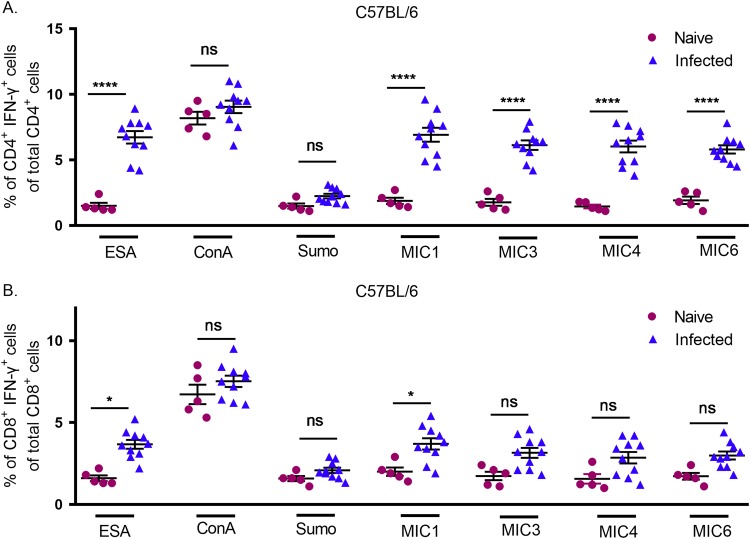
Intracellular cytokine staining of IFN-γ production by CD4^+^ and CD8^+^ T cells from C57BL/6 mice. Splenocytes were obtained from mice that were naive or chronically infected with T. gondii and stimulated with ESA as an antigen-specific positive control (1 µg/ml), ConA as a nonspecific positive control (1 µg/ml), Sumo as a negative control (1 µg/ml), MIC1, MIC4, or MIC6 (1 µg/ml), or MIC3 (0.5 µg/ml) for 24 h. The experiment included 5 naive and 10 chronically infected mice. Ordinary one-way ANOVA with Sidak’s multiple-comparison test was used to compare results between naive and infected cells.

For C57BL/6 mice also, ESA-stimulated splenocytes from infected mice showed higher numbers of CD4^+^ T cells than CD8^+^ T cells among CD3^+^ IFN-γ^+^ cells (Fig. S1B). Stimulation of splenocytes with MIC antigens resulted in significant increases in IFN-γ production by CD4^+^ T cells from infected mice in comparison to those from naive mice (Fig. 4A). In contrast, most MIC antigens, with the exception of MIC1, did not show a significant difference in IFN-γ-producing CD8^+^ T cells (Fig. 4B).

### Analysis of IFN-γ production by memory T cells.

**FIG 5:**
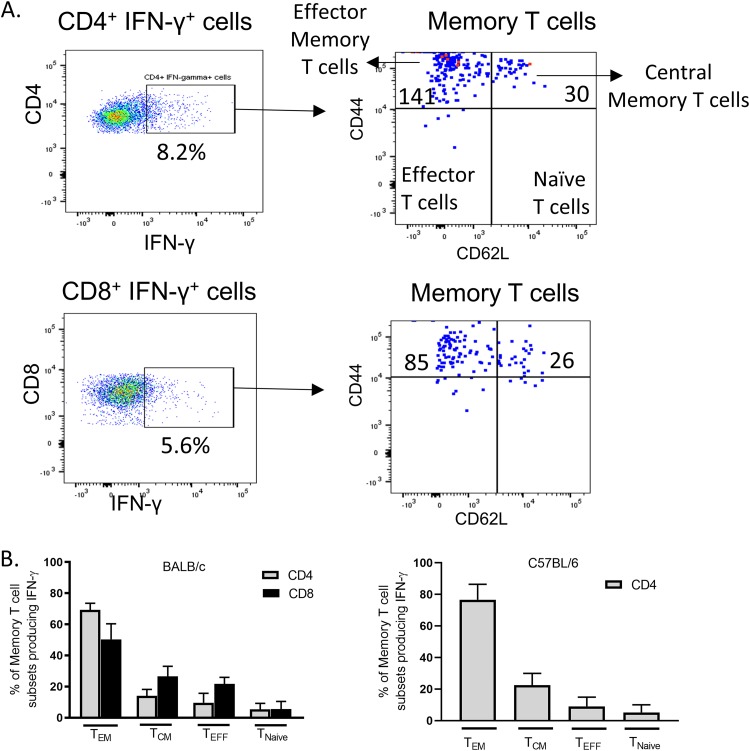
Relative contribution of memory T-cell subsets to production of IFN-γ in response to MIC antigens. (A) The gating strategy for evaluation of CD4^+^ and CD8^+^ memory T-cell subsets producing IFN-γ using CD44 and CD62L markers is shown on the left. The plot on the right present the numbers (shown within the boxes) of CD4^+^ and CD8^+^ memory T-cell subsets producing IFN-γ in splenocytes obtained from BALB/c mice chronically infected with T. gondii when stimulated with 1 µg/ml of MIC1 for 24 h. (B) Frequencies of memory T-cell subsets producing IFN-γ in CD4^+^ and CD8^+^ T cells in response to MIC1 in splenocytes obtained from BALB/c and C57BL/6 mice chronically infected with T. gondii (*n* = 10). Since in C57BL/6 mice IFN- γ was predominantly made by CD4^+^ T cells, memory T-cell analysis was done on CD4^+^ IFN- γ ^+^ T cells.

Memory T cells play a critical role in providing long-term immunity. During secondary challenge, populations of antigen-specific memory T cells have the capacity to mediate vigorous and accelerated responses. However, the relative contributions of different memory subsets (either CD4^+^ or CD8^+^ T cells) to recall responses to T. gondii antigens is unknown. To identify the frequencies of memory T-cell subsets producing IFN-γ in response to MIC antigens, we characterized memory T-cell subsets within CD4^+^ and CD8^+^ T cells. Figure 5A shows the gating strategy used for analyzing the four different T-cell populations, based on expression of cell surface markers CD44 and CD62L. We classified cells based on the expectations that central memory T cells are CD44^hi^ CD62L^hi^, effector memory cells are CD44^hi^ CD62L^lo^, effector T cells are CD44^−^ CD62L^lo^, and naive cells are CD44^−^ CD62L^hi^. For BALB/c mice, memory T-cell subset analysis was done both on CD4^+^ IFN-γ^+^ and CD8^+^ IFN-γ^+^ cells. Since in C57BL/6 mice IFN-γ was predominantly made by CD4^+^ T cells, memory T-cell subset analysis was done only on CD4^+^ IFN-γ^+^ T cells. We found that both in IFN-γ-producing CD4^+^ and CD8^+^ T cells in BALB/c mice and in IFN-γ-producing CD4^+^ T cells in C57BL/6 mice, Tem cells (CD44^hi^ CD62L^lo^) were the predominant cells that contributed to IFN-γ production in response to MIC1 antigen (Fig. 5B). Similar results were obtained with MIC3, MIC4, and MIC6 (data not shown). Although the total number of memory cells was low, approximately 70 to 85% of CD4^+^ IFN-γ^+^ T cells and 58 to 70% of CD8^+^ IFN-γ^+^ T cells were CD62L^lo^ (Fig. 5B). These findings indicate that MIC antigen-experienced CD4^+^ and CD8^+^ T cells were maintained as effector memory cells in mice chronically infected with T. gondii.

## DISCUSSION

Excretory secretory antigens (ESA) of T. gondii play a key role in stimulation of antibody responses the host immune system during T. gondii infection (42). ESA also elicit strong delayed-type hypersensitivity reactions in humans, as detected by skin test, yet the antigens responsible for this response remain unknown (30, 43). Previous mass spectrometry studies done in our lab revealed ESA to be highly enriched in microneme proteins (29). Moreover, the secretory MIC1, MIC3, MIC4, and MIC6 proteins have been shown to induce protective immunity against T. gondii in the murine model (33, 34). In this study, we focused on the role of these secretory antigens in inducing immunological memory during chronic infection in the mouse. Our findings indicate that MIC proteins (MIC1, MIC3, MIC4, and MIC6) elicit cellular immune responses characterized by effector memory T cells that are activated to secrete IFN-γ. These findings suggest that MIC antigens may be useful for developing diagnostic methods that detect cell-mediated responses based on IFN-γ secretion.

Currently diagnosis of chronic toxoplasmosis is based almost entirely on antibody responses where chronic infections are best established based on IgG, while early infection is monitored by IgM (44–49). Although standard serological tests provide excellent assessment of prior infection status, including discrimination between recently acquired and long-term infections, they do not provide an assessment of immunological responsiveness. As well, serological testing in newborns is complicated by the potential for transfer of maternal IgG antibodies. Consequently, it has been shown that testing of cell-mediated immunity in assays, such as detection of IFN-γ release by peripheral blood leukocytes, can provide greater discrimination of infection status in newborns (50, 51). These studies relied on whole parasite antigen, which performed well, but the authors commented that identification of specific antigens may improve test performance (50). In a very different patient population, IFN-γ-specific ELISPOT assays were used to estimate the risk of toxoplasmic encephalitis relapse in patients infected with HIV-1 and on highly active antiretroviral therapy (HAART) (49). Cell-mediated responses to T. gondii antigens in such patients may be a better predictor of the potential for reactivation of central nervous system (CNS) disease versus total CD4 levels that are normally monitored to assess immune status (49). This study was also based on using whole T. gondii soluble antigens from tachyzoites to detect immune status, and greater discrimination might be achieved using specific antigens.

Detection of IFN-γ secretion offers an independent means to assess prior infection, a method that has gained popularity recently for other chronic infections, such as tuberculosis (52–54). An IFN-γ release assay known as T-SPOT TB and based on an ELISPOT test is one of the most sensitive methods used for detecting tuberculosis infection. This method is based on the ability of specialized immune cells (dendritic cells and macrophages) to present antigen on MHC class II receptors to memory T cells, which, in turn, produce IFN-γ. Previous studies using IFN-γ release assays to monitor IFN-γ secretion in T. gondii-infected mice indicate that responses to several GRA and ROP secretory antigens are detected very early after infection (55). Additionally, it has been shown that the parasite proteins GRA1 and SAG1 elicit production of IFN-γ from peripheral blood cells of chronically infected women (56). These findings suggest that it may be possible to develop assays to monitor IFN-γ release in response to specific T. gondii proteins as an alternative to serological assays for detecting both acute and chronic infections.

In this study, we developed an IFN-γ release assay based on the ELISPOT assay to quantify T-cell responses to recombinant MIC antigens found in ESA. Highly specific responses were detected from T cells from infected BALB/c and C57BL/6 mice stimulated with MIC1, MIC3, MIC4, and MIC6, suggesting that conserved MHC class II (MHC-II) epitopes are contained in these antigens. Based on ROC curve analyses, all four MIC antigens displayed very high sensitivity (100%) and specificity (86 to 100%). Of the MIC antigens tested, MIC1 showed the best combination of sensitivity and specificity. Hence, the IFN-γ ELISPOT assay using MIC1 or other antigens may be useful in detecting toxoplasma infection in other animals as well.

Although we have not tested ROC using crude ESA, the use of recombinant antigens offers several advantages. First, by using recombinant antigens, the production of material can be easily scaled and quality controlled from batch to batch. Second, it is evident from deconvolving the mixture of proteins found in ESA that not all antigens are capable of eliciting IFN-γ secretion responses. Hence, using purified recombinant antigens increases the specific activity of the assay. Finally, use of recombinant antigens may also allow avoidance of cross-reactive antigens, which would confound sensitivity and specificity performance.

Although our findings show promise for detecting infection in mice, there are several additional challenges that will need to be met if these studies are to be extended to other animals. First, it will be important to assess the degree to which MIC antigens are recognized across a broader panel of MHC-II receptors from different species. Second, the performance of the recombinant antigens described here may differ in sensitivity and/or specificity in the case of other animals, including humans. Finally, the performance of purified recombinant antigens as a means to detect infection based on IFN-γ stimulation may differ in acute infection versus chronic and for individuals of different immune statuses. Our studies stand as a benchmark for such future studies aimed at extending the utility of these antigens for diagnosis in other species, including humans.

During chronic T. gondii infection, long-term immunity is mainly mediated by T-cell production of IFN-γ required for controlling parasite reactivation (8, 57). Consequently, we also tested the ability of these four MIC antigens to induce IFN-γ production by CD4^+^ and CD8^+^ T cells in T. gondii-infected mice. In BALB/c mice, IFN-γ was found to be produced by both CD4^+^ and CD8^+^ T cells, although responses were higher in CD4^+^ T cells. This observation is in line with another study, in which CD4^+^ T cells were reported to produce more IFN-γ than did CD8^+^ T cells among splenocytes from chronically infected BALB/c mice stimulated with tachyzoite lysate antigen (58). We found that IFN-γ was primarily produced by CD4^+^ T cells in C57BL/6 mice. These results corroborate those of a previous study that reported CD4^+^ T cells as the main IFN-γ-producing cells among splenocytes obtained *ex vivo* from C57BL/6 mice immunized *with* T. gondii (59). The ability of MIC antigens to elicit IFN-γ secretion for both subtypes is consistent with previous findings indicating that these antigens can drive protective immune responses in the mouse (33–35).

To decipher the phenotypes of memory T-cell subsets involved in IFN-γ production in response to MIC antigens, we characterized CD4^+^ and CD8^+^ T cells using phenotypic markers. We chose a time point of 4 weeks postinfection in order to avoid T-cell exhaustion, which has been described in other studies (60). Both CD4^+^ and CD8^+^ T cells producing IFN-γ in response to MIC antigens were found to be almost exclusively CD62L^lo^ cells, suggesting the presence of an extended pool of effector memory cells that produce IFN-γ in a recall response in T. gondii chronic infection. This result is consistent with the effector function of Tem cells, which, although short-lived (61), contribute to IFN-γ production and protection against chronic intracellular parasitic infections with Plasmodium chabaudi and Trypanosoma cruzi in the mouse (62–64).

Taken together, our data demonstrate that MIC antigens found in ESA induce T memory recall responses that lead to IFN-γ secretion in animals chronically infected with T. gondii. Monitoring such responses may provide an adjunct to existing serological tests either to establish infection or to monitor immune responsiveness. Our findings also suggest that MIC antigens induce IFN-γ-producing CD4^+^ and CD8^+^ memory T cells that are maintained and activated against a secondary challenge; hence, these antigens might be exploited for development of protective vaccines.

## MATERIALS AND METHODS

### Ethics.

Animal studies were conducted according to the U.S. Public Health Service policy on human care and use of laboratory animals. Animals were maintained in facilities approved by the Association for Assessment and Accreditation of Laboratory Animal Care (66). Animal studies were approved by the Institutional Animal Studies Committee at the School of Medicine, Washington University, St. Louis, MO.

### Parasite culture and preparation of ESA.

*Toxoplasma* strain RH tachyzoites were maintained in human foreskin fibroblasts as previously described (65). Excretory secretory antigens (ESA) were prepared as described previously, with slight modification (29). Parasites were harvested from fully egressed cultures and resuspended in extracellular (EC) buffer (5 mM KCl, 142 mM NaCl, 1 mM MgCl_2_, 1.8 mM CaCl_2_, 5.6 mM d-glucose, 25 mM HEPES [pH 7.4]). Tissue culture-grade 24-well plates were coated with 1% bovine serum albumin (BSA) in EC buffer at 4°C overnight. The next day, the wells were washed with EC buffer to remove BSA just prior to stimulating microneme secretion. For stimulation of ESA secretion, 500 µl of freshly harvested parasites was added to BSA-coated wells and 500 µl of 1 mM zaprinast (Sigma-Aldrich) in EC buffer was added. Parasites were allowed to secrete for 20 min at 37°C. After 20 min, plates were chilled for 5 min, the liquid was collected and centrifuged at 2,700 rpm and 4°C for 5 min, and the supernatant was retained as the ESA fraction. The ESA fraction was buffer exchanged with phosphate-buffered saline (PBS) using Amicon (Millipore Sigma) centrifugal filters (3-kDa cutoff), and the retained fraction was stored at −80°C. The protein concentration was estimated using a bicinchoninic acid (BCA) protein assay kit (Thermo Fisher).

### Cloning, expression, and purification of MIC proteins.

MIC1, MIC3, and MIC4 constructs and M2AP, MIC6, and MIC10 full-length genes were amplified by PCR using T. gondii ME49 strain cDNA as the template with specific forward and reverse primers (Table S1). After treatment with the BsaI and XbaI restriction enzymes, PCR products were cloned into the pE-Sumo vector and transformed into competent E. coli DH10B. Positive clones were identified using colony PCR screening and confirmed by DNA sequencing. For expression of the Sumo fusion proteins, pE-Sumo-fusion plasmids were isolated from the DH510B cells and chemically transformed into E. coli BL21(DE3) Rosetta cells. A single pE-Sumo-positive colony was inoculated into TB broth with 100 µg/ml of ampicillin until the optical density reached between 0.6 and 0.8, and the culture was induced with 0.5 mM isopropyl-β-d-thiogalactopyranoside (IPTG) at 15°C overnight. Cells were harvested and the cell pellet was resuspended in lysis buffer (Sigma-Aldrich) containing lysozyme and protease inhibitor cocktail. Recombinant MIC proteins were purified using an HIS-Select nickel affinity gel (Sigma-Aldrich) using different concentration of imidazole. The purity of eluted proteins was analyzed by 12% SDS-PAGE electrophoresis and staining with Instant Blue Coomassie stain (Expedeon). Recombinant proteins were dialyzed against PBS (pH 7.2) and treated with Pierce high-capacity endotoxin removal resin (Thermo Fisher) for 8 to 12 h according to the manufacturer’s instructions. The recombinant MIC proteins were quantified by separation on SDS-PAGE gels, staining with Instant Blue Coomassie stain, and comparison with a standard curve of known concentrations of BSA. Samples were then stored at −80°C until use.

10.1128/mSphere.00711-18.2 TABLE S1 List of the primers used in the study. Download Table S1, DOCX file, 0.1 MB. Copyright © 2019 Saraav et al. 2019 Saraav et al. This content is distributed under the terms of the Creative Commons Attribution 4.0 International license.

### Experimental design for *in vivo* infections.

Experiments were performed on 8-week-old specific-pathogen-free (SPF)-grade female BALB/c and C57BL/6 mice purchased from Jackson Laboratory. Studies were approved by Division of Comparative Medicine, Washington University. Animals were maintained in an AAALAC-approved animal facility. For chronic infection, mice were infected by oral administration of 5 to 10 cysts of T. gondii type II strain ME49. Enzyme-linked immunosorbent assay (ELISA) was performed using serum to confirm that the mice were infected. At 30 days postinfection, splenocytes were collected and stimulated with antigens.

### Preparation of mouse splenocytes.

Spleens from naive and T. gondii-infected mice were harvested and splenocytes were released by grinding the spleen through a 70-μm-pore-size nylon cell strainer. Splenocytes were centrifuged at 400 × *g* for 10 min at 4°C, and red blood cells (RBCs) were removed using RBC lysis buffer (Biolegend) for 2 min on ice. Splenocytes were then washed in sterile PBS and Hanks’ balanced salt solution (HBSS; Corning). Cells were quantitated using a hemacytometer. For ELISPOT assay, splenocytes were resuspended in CTL medium (Immunospot) supplemented with 100 U/ml of penicillin and 100 μg/ml of streptomycin (M&C Gene Technology). For IFN-γ intracellular (cytokine staining) and phenotypic characterization, splenocytes were resuspended in RPMI 1640 medium (Gibco) and 10% heat-inactivated fetal bovine serum (HyClone).

### IFN-γ ELISPOT assay.

Briefly, 2.5 × 10^5^ splenocytes per well were stimulated in precoated wells of polyvinylidene difluoride (PVDF) strip plates (Immunospot) with medium alone, ESA (1 μg/ml) for a positive control, concanavalin (ConA; Sigma-Aldrich; 1 μg/ml) for a nonspecific-T-cell positive control, Sumo (1 μg/ml) for a negative control, or different concentrations of MIC antigens (MIC1, 1 μg/ml; M2AP, 1 μg/ml; MIC3, 0.5 μg/ml; MIC4, 1 μg/ml; MIC6,1 μg/ml; and MIC10, 1 μg/ml) for 24 h at 37°C and 5% CO_2_. After the plate was washed and developed according to the manufacturer’s instructions, the antigen recall response was determined by counting the number of spots (IFN-γ-producing cells) per well per treatment. The numbers of IFN-γ-producing T cells following stimulation with T. gondii antigens were detected and calculated using an ELISPOT reader (Immunospot S6 Core; CTL). Receiver operating characteristic (ROC) curves were used to evaluate the accuracy of the ELISPOT assay. Specificity, sensitivity, and cutoff values for MIC1, MIC3, MIC4, and MIC6 were determined for both BALB/c and C57BL/6 mice using the ROC curves.

### Intracellular cytokine staining and immunophenotypic characterization.

Briefly, 2 × 10^5^ splenocytes per well were stimulated with concentrations of MIC antigens similar to those used in the previous experiment. After stimulation, cells were treated with GolgiStop protein transport inhibitor (1 μl/ml; BD Biosciences) for 6 h. Cells were washed and stained with 5 μl of LIVE/DEAD Aqua (Invitrogen) and 50 μl of anti-mouse CD3-allophycocyanin (APC)-Cy7, CD4-peridinin chlorophyll protein (PerCP)-Cy5.5, CD8-fluorescein isothiocyanate (FITC), CD44-phycoerythrin (PE), and CD62L-APC antibodies (Biolegend, USA) prepared in PBS plus 0.5% BSA for 30 min at 4°C in the dark. Cells were fixed and permeabilized with 200 μl of 1× BD Cytofix/Cytoperm buffer for 20 min at 4°C and then washed with 200 μl of 1× BD Perm/Wash buffer. Next cells were incubated with 50 μl of anti-mouse IFN-γ–PE–Cy7 intracellular staining antibody (Biolegend) or anti-mouse PE-Cy7 IgG1, ĸ isotype control (Biolegend), for 30 min at 4°C in the dark. Cells were washed and finally resuspended in 1× BD Perm/Wash buffer. Data analysis was carried out using FlowJo v10 software. Events were gated on singlets, live cells, and lymphocytes using forward scatter and side scatter. Analysis was done using isotype-matched controls as a reference. To identify the frequencies of memory T-cell subsets producing IFN-γ in response to MIC antigens in an *ex vivo* assay, gated CD4^+^ IFN-γ^+^ and CD8^+^ IFN-γ^+^ T cells were evaluated using CD44 and CD62L markers. Cells were acquired using a BD LSRII cytometer (BD Biosciences).

### Statistical analyses.

Data were analyzed in Prism using the D’Agostino and Pearson test to confirm that they were normally distributed. Ordinary one-way analysis of variance (ANOVA) was used to compare ELISPOT results and IFN-γ-positive staining between naive and infected cells. *P* values of *≤*0.05,* ≤*0.01,* ≤*0.001, and* ≤*0.0001 were considered statistically significant. The analyses were done by GraphPad Prism 7.0 software. ROC curves were used to evaluate the accuracy of the ELISPOT assay. The cutoff value was determined from the ROC curve by choosing the value that gave the best sensitivity and specificity.
