# Supplementary figures and images for: Secretory Microneme Proteins Induce T-Cell Recall Responses in Mice Chronically Infected with Toxoplasma gondii
Source: mSphere. 2019 Feb 27;4(1):e00711-18. doi: 10.1128/mSphere.00711-18 (PMC6393730; doi:10.1128/mSphere.00711-18)

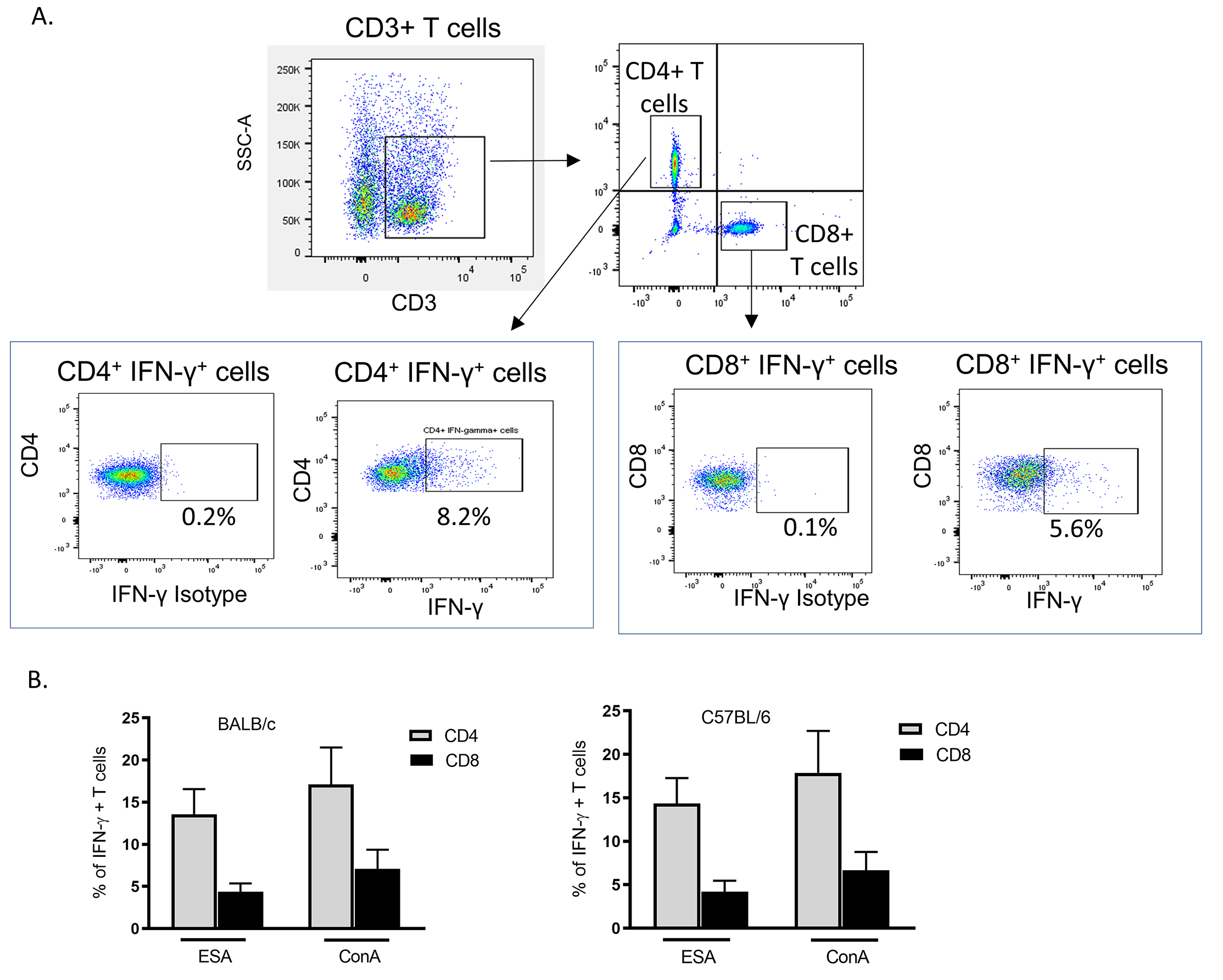

Supplement: FIG S1 [file mSphere.00711-18-sf001.tif]
