# Supplementary material for: Secretory Microneme Proteins Induce T-Cell Recall Responses in Mice Chronically Infected with Toxoplasma gondii
Source: mSphere. 2019 Feb 27;4(1):e00711-18. doi: 10.1128/mSphere.00711-18 (PMC6393730; doi:10.1128/mSphere.00711-18)
Supplement: TABLE S1 [file mSphere.00711-18-st001.docx]

Table S1. List of the primers used in the study

| Name of the antigen | Primers |
| --- | --- |
| MIC1 | F.P – ACT GTG GTC TCT AGG TAT GGA AGC ATA TGG AGA AGC GTC GCA TTC TCA  R.P - ACT GTT CTA GAT CAG AGC GTT AGT TGC TGC CCC TCT TCG AGC GTC GCT T |
| M2AP | F.P- ACT GTG GTC TCT AGG TAT GAG GAA AGT TGG AAA TCC GGC GGC GCA  R.P- ACT GTT CTA GAT TAC GCC TCA TCG TCA CTC GGC AGA |
| MIC3 | F.P – ACT GTG GTC TCT AGG TAT GAT CTA CCA TCC TGA CAA AAG CTA TGG AGG AGA CT  R.P – ACT GTT CTA GAT CAG AGA CTG GCT GGG TAC CTT CGT CT |
| MIC4 | F.P – ACT GTG GTC TCT AGG TAT GAG TTC GGA GCC TGC AAA ACT TGA TCT CTC TTG TGT  R.P – ACT GTT CTAGAT CAG CCA CTG ATC TGA TCT TCC ATC TCC TCT TGA GT |
| MIC6 | F.P – ACT GTG GTC TCT AGG TAT GTC CCC GTT TTT TGC CTT TCT TCC TG  R.P – ACT GTT CTA GAT TAA TCC CAT GTT TTG CTA TCC AAA TCA |
| MIC10 | F.P- ACT GTG GTC TCT AGG TAT GGC GCT TTC TTC TTT GAA CAA TA  R.P- ACT GTT CTA GAT CAC ATT GAT TTC CTG CGT CTT GCG AG |
| Sumo | F.P – ACG GTA TTA GAA TTC AAG CTG ATC A  R.P – CTT TCG GGC TTT GTT AGC AGC |
